# Electronic properties and surface reactivity of SrO-terminated SrTiO_3_ and SrO-terminated iron-doped SrTiO_3_

**DOI:** 10.1080/14686996.2018.1440136

**Published:** 2018-03-02

**Authors:** Aleksandar Staykov, Helena Tellez, John Druce, Ji Wu, Tatsumi Ishihara, John Kilner

**Affiliations:** ^a^ International Institute for Carbon-Neutral Energy Research (WPI-I2CNER), Kyushu University, Fukuoka, Japan; ^b^ Faculty of Engineering, Department of Applied Chemistry, Kyushu University, Fukuoka, Japan; ^c^ Department of Materials, Imperial College London, London, UK

**Keywords:** DFT, perovskites, surface chemistry, oxygen reduction, 50 Energy Materials; 207 Fuel cells / Batteries / Super capacitors, 401 1st principle calculations

## Abstract

Surface reactivity and near-surface electronic properties of SrO-terminated SrTiO_3_ and iron doped SrTiO_3_ were studied with first principle methods. We have investigated the density of states (DOS) of bulk SrTiO_3_ and compared it to DOS of iron-doped SrTiO_3_ with different oxidation states of iron corresponding to varying oxygen vacancy content within the bulk material. The obtained bulk DOS was compared to near-surface DOS, i.e. surface states, for both SrO-terminated surface of SrTiO_3_ and iron-doped SrTiO_3_. Electron density plots and electron density distribution through the entire slab models were investigated in order to understand the origin of surface electrons that can participate in oxygen reduction reaction. Furthermore, we have compared oxygen reduction reactions at elevated temperatures for SrO surfaces with and without oxygen vacancies. Our calculations demonstrate that the conduction band, which is formed mainly by the d-states of Ti, and Fe-induced states within the band gap of SrTiO_3_, are accessible only on TiO_2_ terminated SrTiO_3_ surface while the SrO-terminated surface introduces a tunneling barrier for the electrons populating the conductance band. First principle molecular dynamics demonstrated that at elevated temperatures the surface oxygen vacancies are essential for the oxygen reduction reaction.

## Graphical abstract

**Figure:**
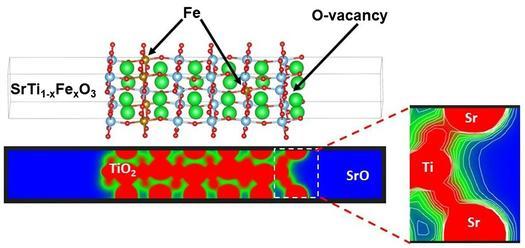

## 1. Introduction

Complex oxide, ceramic materials have attracted significant academic and industrial attention recently with their application in the fields of electronics [1], catalysis, photochemistry [2,3], water electrolysis [4], and solid oxide fuel cells (SOFC). Amongst the various crystal lattices of complex oxides, the perovskites play significant role in modern energy relegated materials’ research. They find application in processes like artificial photosynthesis, steam electrolysis, and most importantly, as SOFC electrodes and electrolytes. The perovskite oxide lattice has the general formula ABO_3_ and it is composed by two different metal ions occupying lattice sites denoted as A-site and B-site. The B-site ions are usually small transition metals characterized with large formal charge, positioned in an octahedral site, coordinated by six oxide ions. Those octahedra are constructing the BO_2_-sublattice of the perovskites. The A-site ions are usually large alkali and alkaline earth metals occupying the cavities formed by the BO_2_-sublattice and neutralizing the charge of the material. However, recent development in the perovskite materials often utilizes rare earth elements from the lanthanoid series as A-site cations in perovskite lattices. Those perovskite materials, containing rare earth elements at the A-sites, are often characterized with supreme catalytic activity [5].

Some A-site or B-site ions can be replaced relatively easily and thus, one can fine-tune the properties of materials. For example, substituents can alter the structural properties of the material or its electronic and optical properties by either changing the band gap width or introducing levels within the band gap. An important consequence of the introduction of dopants is the control on the oxygen stoichiometry in the perovskite lattice. This is achieved by substitution of a lattice cation by cation with lower formal charge. In order to preserve the lattice charge neutrality, oxygen vacancies are formed to compensate the reduced cation charge. While an oxygen vacancy would certainly affect the crystallographic properties of the perovskite lattice and it is likely to affect its electronic properties too, it major purpose would be to facilitate the oxide ion transport through the material. The oxide ion conductivity would be crucial for devises like SOFCs and steam electrolyzers. Oxide ion mobility requires significant activation barrier, hence, the high operating temperature of SOFCs. The oxygen vacancy concentration can be increased by careful doping at the both A-sites and B-sites cations where some dopants are used to stabilize structurally the perovskite lattice [6,7].

Beside oxide ion transport, for successful design of SOFCs or steam electrolyzers one needs fast oxygen reduction reaction on the electrode surfaces. The diatomic oxygen molecule, O_2_, has a triplet spin multiplicity in its ground state and is relatively inert. It can be activated either by thermal excitation (flame), optical excitation to singlet excited state, or electron transfer leading to superoxo species, ${\text{O}}_{2}^{-}$ (one electron transfer), peroxo species ${\text{O}}_{2}^{2-}$ (two electron transfer), or two dissociated oxide ions 2${\text{O}}^{2-}$ (four electron transfer). To improve the oxygen reduction reaction rates on perovskite surfaces, it is necessary to understand the oxygen dissociation mechanism at an atomistic level. Proper surface termination and surface composition are important pre-requirements for understanding the oxygen reduction reaction mechanism. Early first principle studies of oxygen reduction reaction on La_2_NiO_4_ surfaces have determined the importance of the B-site element surface-abundance for high reaction rates [8]. Such a conclusion is in agreement with the high catalytic activity of the transition metals. However, low energy ion scattering (LEIS) experiments have demonstrated that at SOFC operating conditions (elevated temperature) the perovskite surface is characterized by an AO-termination [9–11]. Theoretical studies verified that in perovskites, surface reconstruction can lead to A-site enriched surface and B-site enriched subsurface [2]. The driving force behind this reconstruction is the tendency of the small highly charged B-site ion to build full coordination sphere and thus, to replace the large A-site cation in the subsurface layer. Scanning tunneling microscope images of water adsorbed on strontium ruthenates also report a SrO surface termination [12]. Theoretical and experimental efforts have been made to explain in detail the driving force behind the A-site surface segregation [10,13,14]. Among the different mechanisms were the elastic interactions and the cation charge interaction in the near surface region. The importance of active d-electron rich elements on the perovskite surface was demonstrated by surface decoration with Hf where it could participate in the oxygen reduction reaction as a catalytically active site [15].

The surface activity of AO-terminated perovskites remains an open question due to the presumed inert nature of the A-site cations. They are usually alkali, alkaline earth, and lanthanoid elements with large ionic radii and low population of d-electrons. In a recent study, the catalytic activity of La_2_NiO_4_ has been revealed with first principle theoretical methods [5]. It was shown that the computed charge of the La-ion was significantly different from its formal charge in the perovskite lattice. While it was widely accepted that La has charge of 3+, Bader population analysis of electron density calculated be density functional theory (DFT) method has shown a charge closer to 2+ [16]. The lower charge on La suggests that it partially retains its electron density, which would be available for the building of partially covalent bonds with lattice oxygens [17,18]. On the perovskite surface, that electron density on La-sites would result in dangling bonds that can show significant catalytic activity. Theoretical simulations show that such mechanism is responsible for the relatively low activation barrier for oxygen reduction reaction on the LaO terminated perovskite surfaces [5]. In-depth analysis shows that this electron density is available due to the different ionization potentials of La 6s and 5d electrons.

Due to the lack of d-electrons in the valence shell, the oxygen reduction reaction for perovskites with alkali and alkaline earth elements on their A-site, should proceed with different mechanisms. Static and dynamic first principle simulations have demonstrated that the surface oxygen vacancies should play a key role in the oxygen dissociation reaction [19–21]. First principle calculations demonstrated that surface vacancies in iron doped SrTiO_3_ are energetically stabilized within the surface AO-layer. Furthermore, pairing of oxygen vacancies is energetically favored due to electron density relaxation in the vicinity of subsurface oxygens. Such vacancy pairs provide additional catalytic sites for the dioxygen dissociation [21]. The oxygen dissociation proceeds through molecular oxygen adsorption within a vacancy site. Electron density is transferred through the d-electron states of the subsurface Ti atoms and the delocalized BO_2_ subnetwork. As a result, the oxygen molecule is activated to a superoxo state, which leads to oxygen dissociation. One of the oxygen atoms occupies the vacant lattice site while the second remains as a surface oxygen species which can recombine with lattice oxygen (molecule-surface oxygen exchange) or occupy neighboring lattice vacancy site.

While the mechanism of oxygen dissociation on alkali and earth-alkali perovskites has been revealed, the nature of the catalytic activity of the oxygen vacancy site remains unclear and requires further investigation. In this work, we look in detail in the electronic properties of SrO terminated surfaces of SrTiO_3_ with and without oxygen vacancies. Our goal is to elucidate if the surface oxygen vacancies offer only geometrical, catalytically active sites or they additionally provide electronic density and access to electronic states which are not available on the pristine SrO surface. To do that we investigate the electronic properties of iron doped SrTiO_3_ surfaces with and without vacancies within the surface AO-terminated layer. In all other equal conditions, the only difference in our models is the availability of surface oxygen vacancies. From Ref. [21], we already know that those surface vacancies would be the catalytic sites for molecular oxygen dissociation. However, in this work we provide details on the electronic structure within and in the vicinity of the oxygen vacancy, compared to the unperturbed SrO surface. We provide theoretical evidence that surface oxygen vacancies are the electron-rich surface active sites that can provide electron density for oxygen dissociation. We show that lack of surface oxygen vacancies limits the electron density in the vicinity of the AO-terminated surface which in order will hinder the molecular oxygen dissociation.

## 2. Methods of calculation

Periodic, plane wave DFT calculations and first principle molecular dynamics were performed with the Vienna Ab initio Software Package (VASP) [22–24]. Perdew–Burke–Ernzerhof (PBE) exchange-correlation functional was applied using projector augmented wave pseudopotentials [25]. Electron energies were converged to 10^−5^ eV using tetrahedron smearing method with Bloch correction for bulk systems and Gaussian smearing for surfaces. The calculations were performed with 400 eV cut-off energy and Monkhorst-Pack k-points mesh of 6 × 6 × 4 for the bulk systems and 6 × 6 × 1 for the slab systems. The selected electronic methods would allow us to obtain accurate density of states (DOS) plots. Geometry optimization was performed using the conjugated gradient algorithm. For bulk systems relaxation was performed of the cell volume, cell shape and atomic positions. For slab models relaxation was performed for the atomic positions only. Slabs were constructed using 12 alternating layers of SrO and TiO_2_ in the [0 0 1] crystallographic directions. The coordinates of the atoms in the middle four layers (two SrO and two TiO_2_) were fixed, while the coordinates of the four layers (two SrO and two TiO_2_) at each surface were fully relaxed. In this way, we could investigate the properties of both the SrO-terminated and the TiO_2_-terminated surfaces. The relaxation was performed until the forces converged to values bellow 0.03 eV/Å^2^. While DFT calculations provide excellent agreement with the experiment for metallic systems, as well as, very good trend for the geometry and reactivity of metal oxides and molecular systems, it is well known that the method fails to describe correctly the optical properties of semiconductors and insulators due to underestimating of the energy levels of the conduction band. This problem could be avoided by the use of GGA+U method, which takes into account local correlations within selected orbitals. However, results depend on the value of the on-site Coulomb interaction, as well as, on the set of orbitals for which the corrections are applied. The value of U is selected to match an experimentally observed property, most often the band gap. Such approach introduces empirical term to DFT and deviates it significantly from its first principle nature. A better approach (completely based on first principle methods) would be the use of hybrid DFT functional, which includes a degree of Hartree–Fock mixing. However, hybrid DFT calculations with plane wave basis sets and large unit cells are computationally expensive. That is why in this study we have employed the GGA+U approach for the d-orbitals of Ti and Fe. The value of U for Ti was estimated to be 8.0 eV by reproducing the optical band of SrTiO_3_, 3.2 eV, and comparing with data from the literature [26]. The value of U for Fe, 3.0 eV, is taken from the literature where it was optimized for SrFeO_3_ [27].

First principle molecular dynamics calculations were performed using the VASP software. The simulation time was 1 ps and the time step was 0.5 fs. Simulation temperature was 1073 K, which corresponds to the operating conditions of SOFC electrodes. Lattice expansion was estimated to be 1% for sequential first principle molecular dynamics calculations performed for the bulk unit cell and expansions of 0% (0 K lattice), 0.5, 1, 1.5, 2, and 3%. Throughout this study, we have used the graphical visualization package VESTA [28].

## 3. Results and discussion

**Figure 1.**
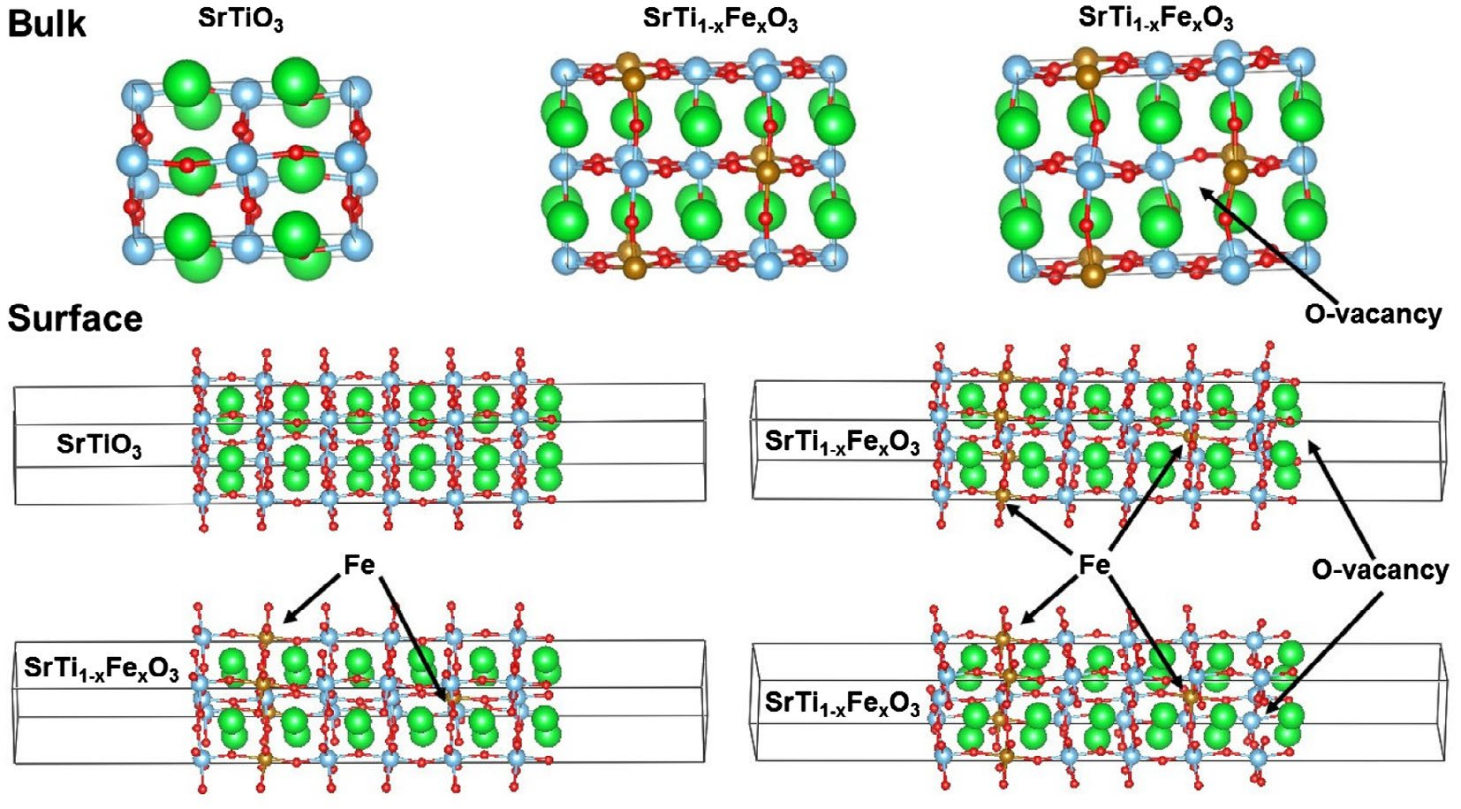
Investigated models of bulk SrTiO_3_, bulk iron-doped SrTiO_3_ without oxygen vacancies, bulk iron-doped SrTiO_3_ with one oxygen vacancy per two iron atoms, SrO terminated surface of SrTiO_3_, SrO terminated surface of iron-doped SrTiO_3_ without oxygen vacancies, SrO terminated surface of iron-doped SrTiO_3_ with oxygen vacancy in the surface layer, and SrO terminated surface of iron doped SrTiO_3_ with oxygen vacancy in the subsurface layer.

In this study, we investigate the DOS and electron density distribution in bulk SrTiO_3_ and SrTiO_3_ surfaces, as well as, high temperature oxygen reduction reaction. We should clearly note that SrTiO_3_ and iron doped SrTiO_3_ differ from the actual operating materials in SOFC such as La_x_Sr_1-x_Co_y_Fe_1-y_O_3-δ_. SrTiO_3_ and iron doped SrTiO_3_ provide easier computational model due to the absence of late transition metals such as Co and heavy rare earth elements, such as La. However, the results provided in this work can be relevant because actual SOFC surface are often SrO terminated [9]. Surface slabs are explicitly modeled with SrO termination as such termination was determined experimentally using LEIS for SrTiO_3_ exposed for short time to elevated temperature [9,21]. For the bulk and the surface calculations three different models were considered: pure SrTiO_3_, iron doped SrTiO_3_ without oxygen vacancies with Fe in 4+ formal charge, and iron doped SrTiO_3_ with one oxygen vacancy per two iron atoms with Fe in 3+ formal charge. In case of surface simulations, oxygen vacancies were considered in the surface SrO layer and the subsurface TiO_2_ layer. Our purpose is to determine the influence of oxygen vacancies and the effect of iron formal charge on SrTiO_3_ band gap and surface electronic states, i.e. contribution to states within the band gap by the surface atoms. In Figure 1 are shown the investigated models of bulk SrTiO_3_, bulk iron doped SrTiO_3_ without oxygen vacancies, bulk iron doped SrTiO_3_ with one oxygen vacancy per two iron atoms, SrO terminated surface of SrTiO_3_, SrO terminated surface of iron doped SrTiO_3_ without oxygen vacancies, SrO terminated surface of iron doped SrTiO_3_ with oxygen vacancy in the surface layer, and SrO terminated surface of iron doped SrTiO_3_ with oxygen vacancy in the subsurface layer.

**Figure 2.**
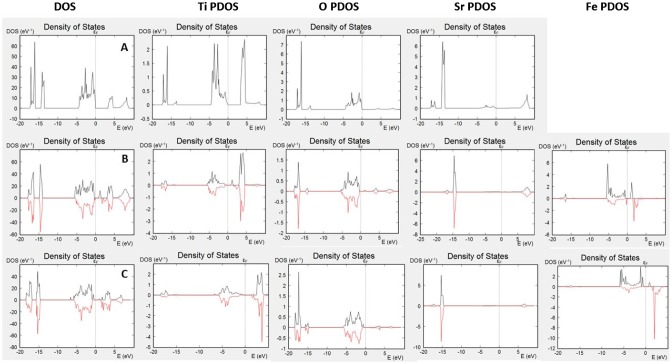
DOS and PDOS of (A) bulk SrTiO_3_; (B) bulk iron-doped SrTiO_3_ without oxygen vacancies; (C) bulk iron-doped SrTiO_3_ with one oxygen vacancy per two iron atoms.

Figure 2(A)–(C) show DOS and partial DOS (PDOS) plots for bulk SrTiO_3_, iron-doped SrTiO_3_ without oxygen vacancies, and iron-doped SrTiO_3_ with one oxygen vacancy per two iron atoms, respectively. The SrTiO_3_ valence band is mainly built by the oxygen 2p atomic orbitals, while the conduction band is formed by the Ti 3d atomic orbitals. Within the conduction band, only a small hybridization of the oxygen orbitals was observed. Sr has no contribution to the DOS in the vicinity of the band gap. Fe^4+^ doping leads to spin polarization of the DOS with both, majority and minority spin levels within the band gap. The conduction band again can be attributed to the Ti 3d-atomic orbitals while the valence band is due to the contribution of the oxygen atoms 2p-atomic orbitals.

The levels within the band gap can be attributed mainly to the Fe^4+^, however, it should be noted that both Ti and O have minor contribution to those levels, i.e. Fe^4+^, affects the electronic properties of the entire BO_2_ sub-lattice. The behavior of the Fe^3+^ doping, shown in Figure 2(C) is different with significant decrease in the Fe-levels within the band gap. Additionally, the contribution to the band gap due to hybridized Ti states is also decreased. The observed DOS spectra in Figure 2 suggest that while Fe^3+^ doping induces only mild changes in the electronic properties of SrTiO_3_, Fe^4+^ doping leads to major changes and multiple levels within the band gap.

**Figure 3.**
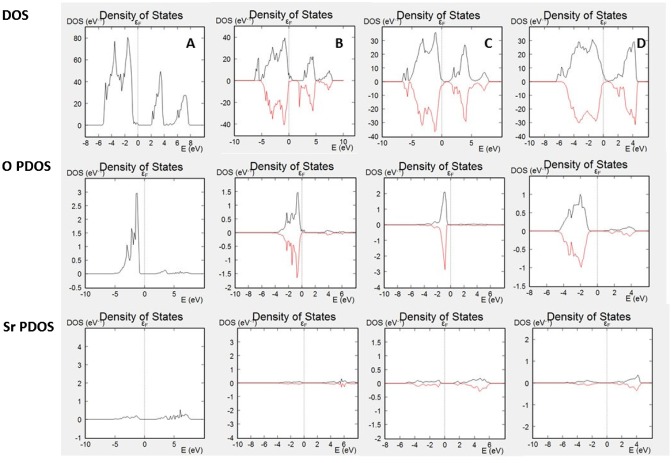
DOS and PDOS of (A) SrO surface of SrTiO_3_; (B) SrO surface of surface iron-doped SrTiO_3_ without oxygen vacancies; (C) SrO surface of iron-doped SrTiO_3_ with oxygen vacancy in the surface layer; (D) SrO surface of iron-doped SrTiO_3_ with oxygen vacancy in the subsurface layer.

The calculations of the bulk electronic properties provide important information for the materials, such as electronic and ionic conductivities, photoelectric properties, optical properties, thermal conductivities, etc., however, they provide only limited information for the surface-related properties, such as catalytic activity for various reactions. In order to understand those processes, we should investigate the electronic properties to several surface layers of the material. Surface states differ from bulk electronic structure by providing additional electron density through dangling bonds or surface reconstruction. In addition, depending on the surface termination, some bands might be missing from the surface spectrum. DOS plots and PDOS plots for SrO terminated slabs of SrTiO_3_, iron doped SrTiO_3_ without oxygen vacancies, and iron doped SrTiO_3_ with one oxygen vacancy per two iron atoms (vacancy on the surface and within the subsurface layer) are shown in Figure 3. Figure 3(A) plots the total DOS of the investigated surface slab of SrTiO_3_ and the PDOS of the surface (only) oxygen atoms and surface (only) Sr atoms. The plots demonstrate that surface oxygen atoms contribute to the valence band but there is no contribution to the conduction band. Sr atoms do not have any significant contribution either to the valence band or to the conduction band as their occupied energy levels are characterized with lower energies and their non-occupied levels are characterized with higher energies. Figure 3(B) plots the total DOS of the investigated surface slab of iron doped SrTiO_3_ without oxygen vacancies and the PDOS of the surface (only) oxygen atoms and surface (only) Sr atoms. While the slab DOS resembles the bulk DOS plotted in Figure 1(B), the SrO surface termination is characterized only with states from the valence band, while the conduction band is not presented on the surface. Same conclusions can be made for iron doped SrTiO_3_ with oxygen vacancies in the surface and subsurface layers plotted in Figure 3(C) and (D), respectively. All investigated slabs are characterized with surface states only from the valence band, while the lower energy edge of conduction band does not propagate to the surface.

**Figure 4.**
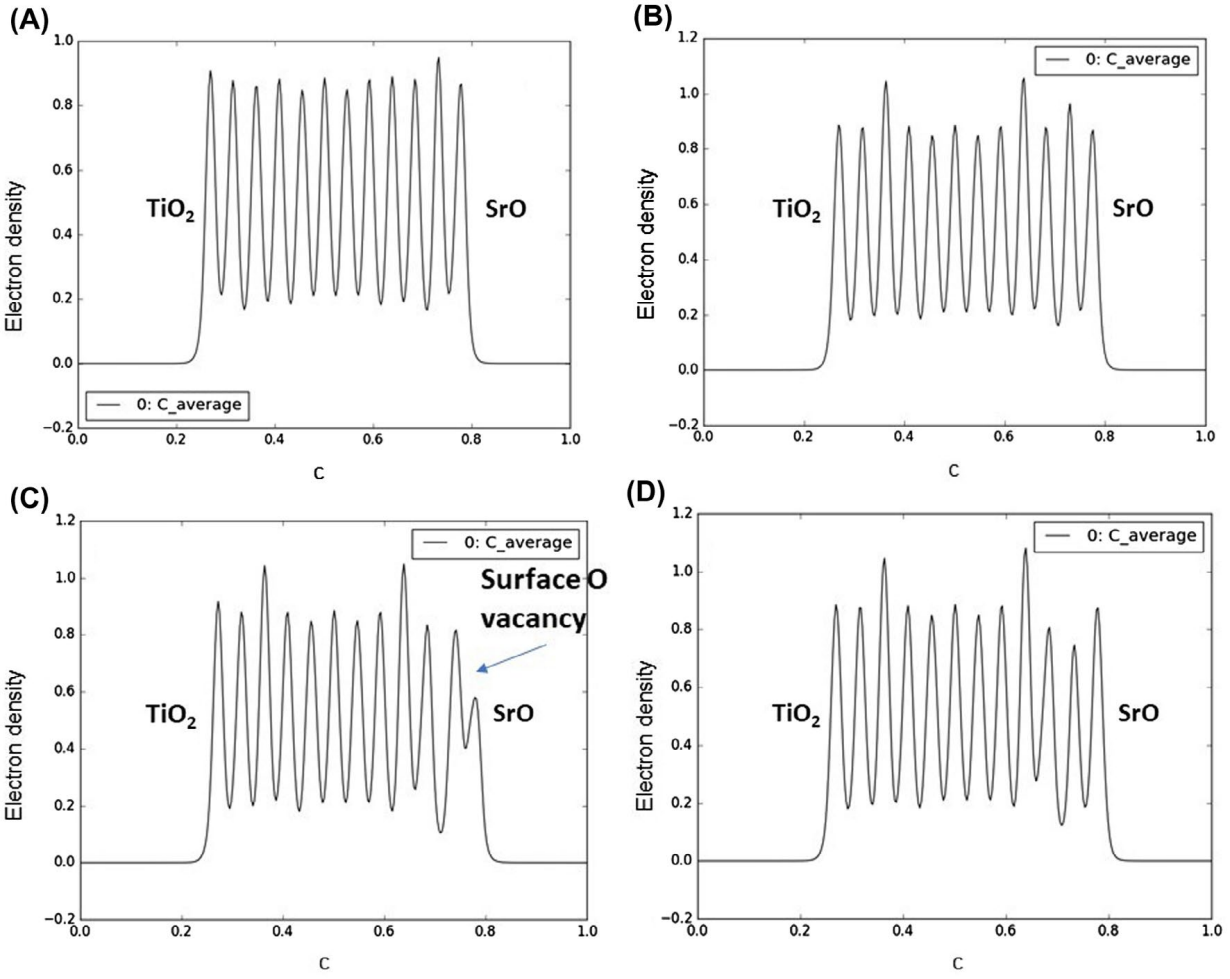
Projected average electron density along the c axis of the slabs for (A) SrTiO_3_ slab; (B) iron-doped SrTiO_3_ slab without oxygen vacancies; (C) iron-doped SrTiO_3_ slab with oxygen vacancy in the surface layer; (D) iron-doped SrTiO_3_ slab with oxygen vacancy in the subsurface layer. Electron density is given in e^−^/Å^3^.

Oxygen reduction reactions on perovskite surfaces are result of surface to molecule electron transfer. Thus, the surface electron density can play an important role for the material’s catalytic activity and the reaction rate. Instead of analyzing the integrated charge density per atom, i.e. Bader population analysis [16], we investigate the overall electron density distribution in the slab along the c axis. Figure 4 shows the average electron density along the c axis for slabs of SrTiO_3_, iron doped SrTiO_3_ without oxygen vacancies, and iron doped SrTiO_3_ with one oxygen vacancy per two iron atoms (vacancy on the surface and within the subsurface layer). For each plot the left surface is TiO_2_ terminated while the right surface is SrO terminated. Our analysis was performed for the SrO terminated surface.

Figure 4(A) shows the average electron density distribution along the c axis for slab of SrTiO_3_. The plot shows that the subsurface TiO_2_ layer is electron enriched compared to the bulk TiO_2_ layers. Figure 4(B) shows the average electron density distribution along the c axis for slab of iron doped SrTiO_3_ without oxygen vacancies. The Fe atoms are located in the 4th and 10th layers. The plot shows that those layers are characterized with increased electron density, hence, the additional electron density of Fe remains localized. Similar to the SrTiO_3_, the subsurface TiO_2_ layer is electron enriched compared to the bulk TiO_2_ layers. Figure 4(C) shows the average electron density distribution along the c axis for slab of iron doped SrTiO_3_ with oxygen vacancy within the surface SrO layer. The plot shows reduced electron density within the surface SrO layer corresponding to the missing oxygen. Unlike the SrTiO_3_ (Figure 4(A)) and iron doped SrTiO_3_ without vacancy (Figure 4(B)), the subsurface TiO_2_ layer is not electron enriched compared to the bulk TiO_2_ layers. However, significant electron density of 0.4 e^−^/Å^3^ can be found in the interlayer space between the surface SrO layer and subsurface TiO_2_ layer. We can associate the additional electron density with spatially directed electron clouds at the subsurface Ti atom exposed at the vacancy site. Figure 4(D) shows the average electron density distribution along the c axis for slab of iron doped SrTiO_3_ with oxygen vacancy within the subsurface TiO_2_ layer. The electron density distribution is similar to that of iron doped SrTiO_3_ without oxygen vacancies (Figure 4(B)) with main difference that the subsurface TiO_2_ layer where the vacancy resides is electron deficient.

**Figure 5.**
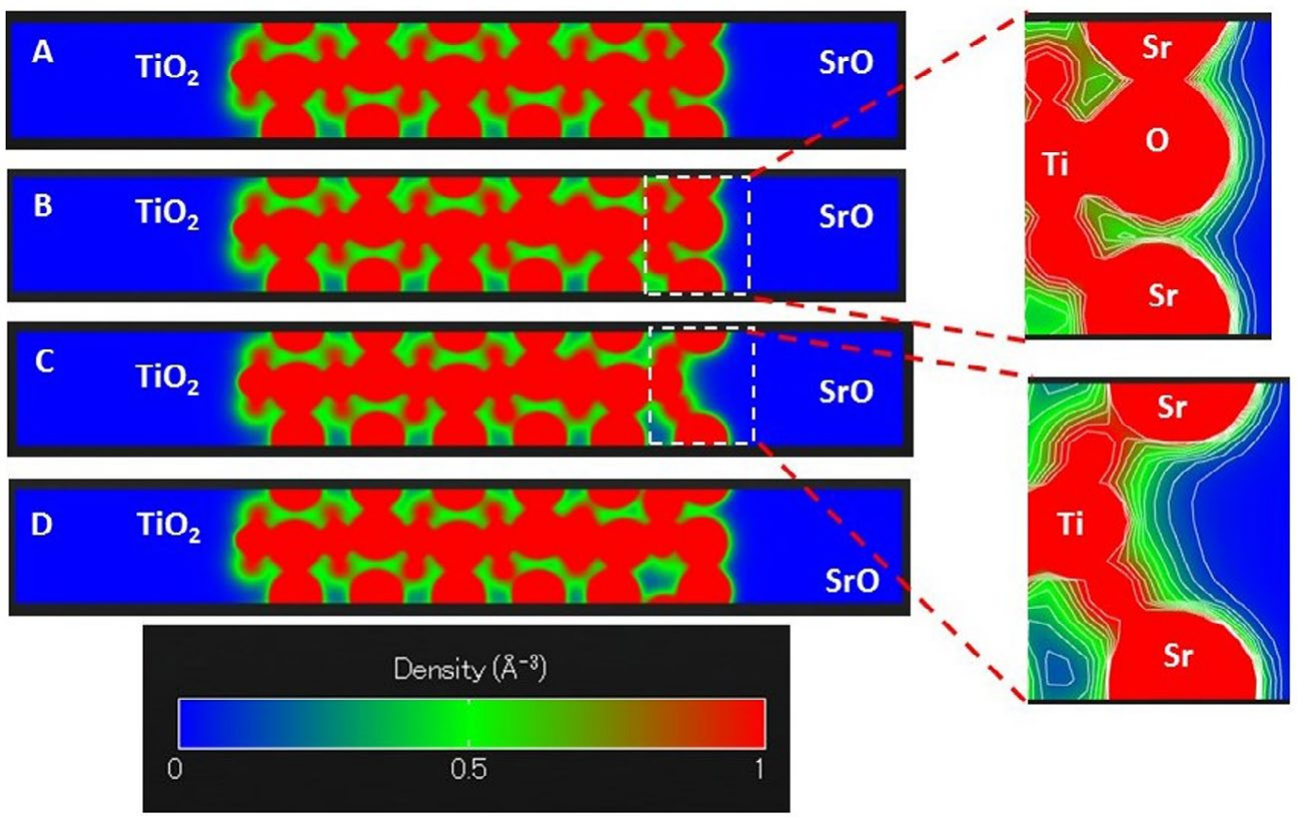
Slice-planes through the electron density along the c axis of the slabs for (A) SrTiO_3_ slab; (B) iron-doped SrTiO_3_ slab without oxygen vacancies; (C) iron-doped SrTiO_3_ slab with oxygen vacancy in the surface layer; (D) iron-doped SrTiO_3_ slab with oxygen vacancy in the subsurface layer. Electron density is given in e^−^/Å^3^. Electron density between 0 and 1 e^−^/Å^3^ is plotted in the blue–green–red color scheme. Contour lines slow the electron density surfaces at each 0.1 e^−^/Å^3^.

To understand better the electron density distribution, we investigate slice-planes along the c axis of the four slabs. The results are shown in Figure 5 where the electron density between 0 and 1 e^−^/Å^3^ is plotted in the blue–green–red color scheme. Contour lines show the electron density surfaces at a step of 0.1 e^−^/Å^3^. The electron density distributions at the SrO terminated surfaces shown in Figure 5(A), (B), and (D), are very similar, that is why we will discuss the differences between the densities of iron doped SrTiO_3_ without the oxygen vacancy (Figure 4(B)) and iron doped SrTiO_3_ with an oxygen vacancy in the surface SrO layer (Figure 4(C)). The slice-plane of Figure 5(C) passes through the center of the vacancy. The contour lines show that 0.1 e^−^/Å^3^ electron density propagates far from the Ti atom and reaches the surface through the oxygen vacancy. Here we should note that the conduction band is localized on the Ti and Fe atoms. Thus, through a surface oxygen vacancy the conduction band can be exposed to the slab surface.

The electron density distribution plotted in Figures 4 and 5 demonstrates that iron doping alone is not sufficient to add electron density to SrO terminated SrTiO_3_ surface. It is the surface oxygen vacancy that exposes subsurface B-sites and provides additional delocalized electron density to the oxygen vacancy cavity. Evidence for such electron density can be seen in Figure 4(C) where it is localized between the surface layer and the subsurface layer. Such electron delocalization pattern was not observed for any of the other models investigated in Figure 4. Furthermore, Figure 5(C) shows that the 0.1 e^−^/Å^3^ electron density contour propagates further from the Ti ion compared to the same contour from Sr ion and O ion in case of Figure 5(B) where surface oxygen vacancy is not considered. Thus, the surface oxygen vacancies should be the key catalytic active sites for the oxygen dissociation reaction. The detailed vacancy facilitated reaction mechanism for oxygen dissociation and lattice incorporation on SrO-terminated, iron doped SrTiO_3_ has been studied in detail in a recent theoretical work [21].

The main disadvantage of DFT is that as a ground state method, thermally excited states are not part of the solution and all calculations are performed for zero Kelvin temperature. In our previous work [21], we have provided reaction mechanism for the oxygen reduction reaction on SrO terminated iron doped SrTiO_3_ slab with oxygen vacancy in the surface layer based on the transition state theory. It is worth noting that the activation barriers obtained with zero Kelvin DFT calculations can be linked to the reaction rate constant and temperature using the Arrhenius equation. An alternative approach is to perform first-principle molecular dynamics where the thermally excited states will be included in the time dependent simulation. Molecular mechanics allows us to include in the simulation different vibronic states and observe the time evolution of the system as a function of its starting geometry and reaction temperature. A disadvantage of first-principle molecular dynamics is its high computational cost. Novel developments such as meta dynamics can significantly reduce the computational efforts.

In this study, we performed high temperature molecular dynamics simulations of oxygen reduction reactions on iron doped SrTiO_3_ slab without oxygen vacancy and iron doped SrTiO_3_ slab with oxygen vacancy in the surface layer. We should note that the effect of a surface oxygen vacancy was already studied with molecular dynamics for SOFC electrodes [20]. In our study, the purpose of the simulation is to observe the relative catalytic activity between the electron rich, Fe^4+^ doped SrTiO_3_ slab and the surface oxygen vacancy as an active site at realistic conditions compared to our previous zero Kelvin simulations [21]. All our simulations are performed for the SrO surface, which was experimentally characterized by LEIS [9,21].

**Figure 6.**
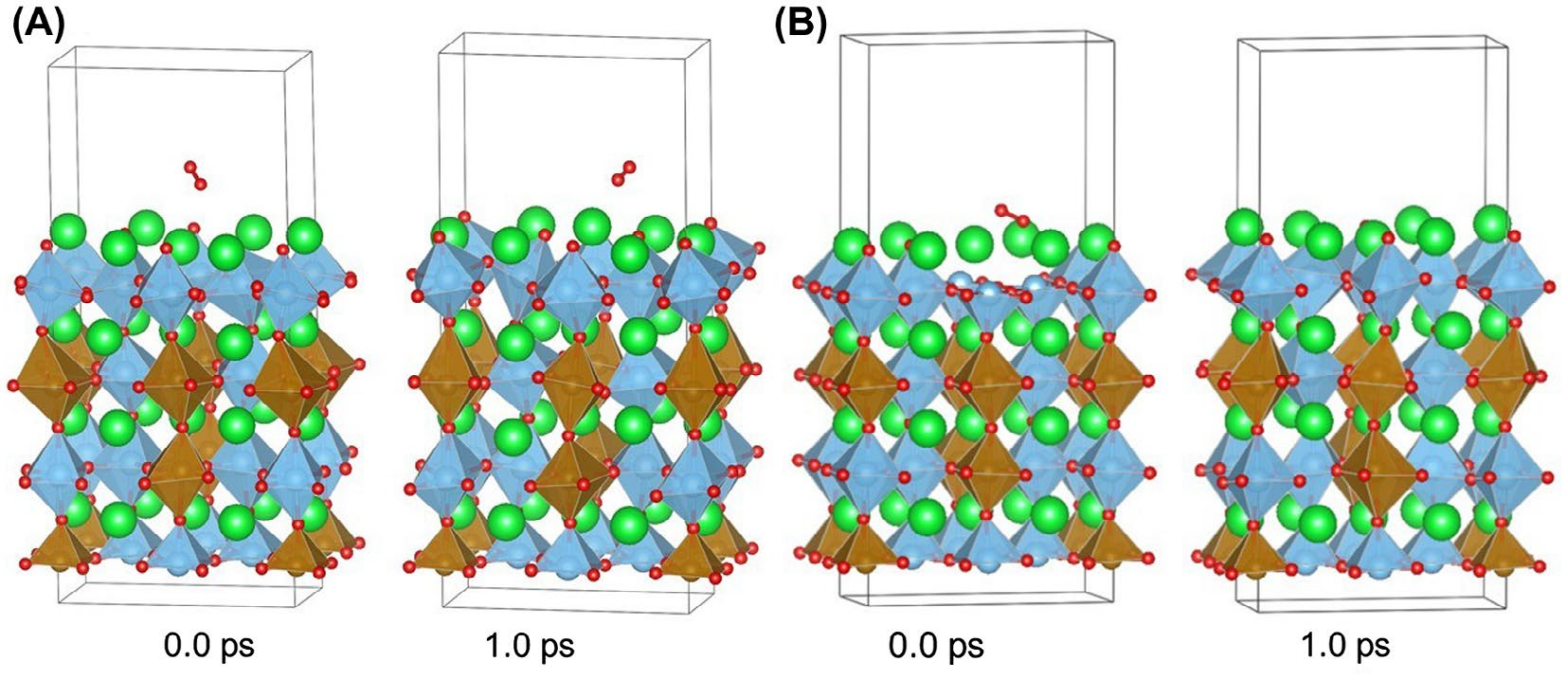
Starting and end geometries for first-principle molecular dynamics simulation at 1000 °C for (A) iron-doped SrTiO_3_ slab without oxygen vacancies and (B) iron-doped SrTiO_3_ slab with oxygen vacancy in the surface layer. Sr is denoted with green color, Ti is denoted with blue color, Fe is denoted with brown color, and O is denoted with red color.

Figure 6 shows the start and end geometries of oxygen molecule interacting with iron doped SrTiO_3_ slab without oxygen vacancies and iron doped SrTiO_3_ slab with oxygen vacancy in the surface layer. The simulations were performed at 1000 °C for simulation time of 1.0 ps. Several runs were performed in order to verify the results. On the iron doped SrTiO_3_ slab without oxygen vacancies in the surface the oxygen molecule has random motion and does not spend significant time over any surface adsorption site. Its behavior does not suggest interaction with the surface and there were no indications for long-lifetime chemisorption which would result in surface to molecule electron transfer.

In the case of the iron doped SrTiO_3_ slab with oxygen vacancies in the surface, two unit cells were considered with neighboring vacancy cites. It was shown that such a configuration with vacancy pairing on the surface is energetically favored due to reduced B-site – oxygen – B-site angles and relaxed electronic density in the vicinity of the lattice oxygens [21]. In our simulation the oxygen molecule was chemisorbed within one of the surface oxygen vacancies and was dissociated over a Sr–Sr bridge. The second oxygen atom occupies the neighboring vacancy site. The oxygen dissociation over the vacancy pair proceeds within 0.4 ps. The reaction mechanism obtained from the high temperature first-principle molecular dynamics is in agreement with the reaction mechanism obtained from the zero Kelvin DFT calculations.

In a recent study, Metlenko et al. [29] have investigated oxygen reduction reaction on iron doped SrTiO_3_ and have claimed that oxygen reduction is solely proportional to the Fe^4+^ content in SrTiO_3_ which would increase the SrTiO_3_ Fermi level. In their study, they have proposed a model for oxygen dissociation including electron transfer to the oxygen molecule involving electrons occupying the conduction band without the assisting function of a surface oxygen vacancy. However, our electron density and DOS analysis summarized in Figures 3–5 demonstrates that the conduction band propagates to the surface only in case of surface oxygen vacancy. In case of high Fe^4+^ content but no surface oxygen vacancy the increased electron density remains within the iron doped SrTiO_3_ subsurface layers. High temperature DFT molecular dynamics allows for the electronic population of low-laying thermally excited states in contrast to the zero K DFT calculations. Even when those thermal excitations were taken into account the surface oxygen vacancies remained the catalytically active sites as demonstrated in Figure 6.

## 4. Conclusions

In this study, we compared the electronic properties of bulk SrTiO_3_ with and without Fe^+3^ and Fe^4+^ doping versus the electronic properties of the corresponding surface slabs with SrO surface termination. Our calculations demonstrate that surface electronic properties deviate significantly from the properties of the bulk materials. In bulk SrTiO_3_ the valence band is formed by the Ti 3p and 3d, and O 2p levels while the conduction band is formed by Ti 3d levels. Sr levels are situated energetically deeper and do not contribute either to the conduction or to the valence bands. Addition of Fe^4+^ doping on the Ti^4+^ sites leads to levels within the band gap. Those levels can clearly contribute to Fe^4+^_,_ however, DOS analysis shows also mixing of the O and Ti levels, e.g. orbital hybridization. Addition of Fe^3+^ doping on the Ti^4+^ sites compensated by one oxygen vacancy per two Fe^3+^ reduces the number of states in the band gap compared to Fe^4+^ doping.

Our study shows, however, that in case of SrO terminated slabs the conduction band is not present at the surface species and that result is independent on the Fe^3+^ /Fe^4+^ doping. Only the valence band is present through surface lattice oxygens. Thus, it is unlikely to have conduction band catalyzed oxygen reduction as proposed by some studies unless the conduction band is exposed to the surface [29]. We show that when surface oxygen vacancies are present in the SrO terminated slab the subsurface Ti atoms are exposed. Those Ti atoms contribute with states to the conduction band. We have also shown that those Ti atoms provide electron density to the surface through the oxygen vacancy. Finally, we have demonstrated using high-temperature first principle molecular mechanics that Fe doping of SrTiO_3_ is not sufficient for oxygen reduction reaction. We have shown that surface oxygen vacancies are the active catalytic sites for the molecular oxygen dissociation.

## Disclosure statement

No potential conflict of interest was reported by the authors.

## Funding

This work was supported by World Premier International Research Center Initiative (WPI), Ministry of Education, Culture, Sports, Science, and Technology of Japan (MEXT), Japan, JSPS, Japan and the NSF, US, under the JSPS-NSF Partnerships for International Research and Education (PIRE).
